# Exploring factors influencing machine milk yield of dairy cows in cow-calf contact systems: Cow behavior, animal characteristics, and milking management

**DOI:** 10.3168/jdsc.2023-0480

**Published:** 2024-04-20

**Authors:** Heather W. Neave, Emma Hvidtfeldt Jensen, Amelie Solarino, Margit Bak Jensen

**Affiliations:** Department of Animal and Veterinary Science, Aarhus University, Tjele 8830, Denmark

## Abstract

The objective of this study was to explore factors contributing to low machine milk yield of cows with cow-calf contact. Danish Holstein dairy cows were assigned to 1 of 3 cow-calf contact treatments: full-time with the calf (n = 24), part-time with the calf between morning (a.m.) and afternoon (p.m.) milking and separated overnight (n = 23), or separated from the calf at birth (n = 23). Behavior of cows (stepping and kicking in the parlor) and milking procedures (teat or udder stimulation frequency, preparation duration) were recorded at a.m. and p.m. milking on 2 d during wk 4 and 6 of the treatment period (8 milkings per cow). Full-time cows had the lowest machine milk yields (mean ± SE; 6.1 and 6.0 ± 0.7 L at a.m. and p.m. milking, respectively), whereas part-time cows had similar yield to no-contact cows at a.m. milking (19.6 ± 0.7 L) and similar yield to full-time cows at p.m. milking (6.4 ± 0.7 L). Lower machine milk yields in cows with calf contact could not be fully attributed to milk consumption by the calf; however, they also could not be explained by differences in cow parity, calf sex, milking procedures (stimulation frequency; preparation duration), or stepping and kicking (restless) behavior at milking. All cows showed more restless behavior at p.m. milking, which weakly negatively affected machine milk yield, but this was unrelated to stimulation frequency in cow-calf contact cows, indicating that there may be other sources of discomfort or stress at the time of milking for these cows. Part-time versus full-time calf contact improved daily machine milk yields, but low machine milk yield issues merit further research so that cow-calf contact systems can be sustainable for producers.

## Highlights

• Cows with full-time calf contact had lower-than-expected machine milk yields.
• Parity, calf sex, milking procedures, and cow restlessness did not explain full-time cows' low yields.
• Part-time calf contact improved milk yield at morning milking.
• Restlessness was unrelated to stimulation frequency.
• A further understanding of factors affecting machine milk yield in cow-calf systems is needed.

## Graphical Abstract

**Figure:**
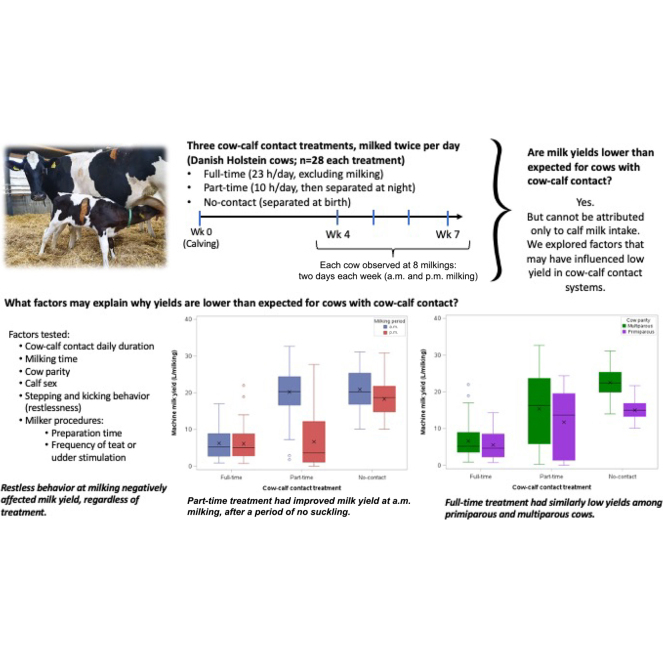
**Summary** We explored factors affecting milk yield of cows with full- and part-time cow-calf contact, compared with cows with no calf contact. Full-time cows had the lowest milk yields, which were similar to part-time cows' yield at afternoon milking after their 10-hour cow-calf contact period. These low yields could not be fully attributed to milk consumption by the calf. Parity, calf sex, milking procedures, and cow restlessness could not explain these lower-than-expected yields. The milk yield of part-time cows did not differ from that of no-contact cows after overnight separation from their calves. Restlessness was greater in all cows at afternoon versus morning milking but was unrelated to stimulation frequency in full- and part-time cows, indicating that restlessness may be due to other discomforts at milking, or stress. The reasons for low milk yield in cow-calf contact systems, apart from milk consumption by the calf, remain to be understood.

There is growing interest by farmers and other stakeholders to implement alternative housing systems for dairy cattle, where contact between cow and calf is prolonged (Eriksson et al., 2022; Neave et al., 2022a). However, a major concern from the dairy producer's perspective is the loss of salable milk and milk ejection issues (Johnsen et al., 2016; Vaarst et al., 2020; Neave et al., 2022a) in dam-rearing systems. Reduced milk yields are expected in cows that nurse (Zipp et al., 2018; Barth, 2020), but if yields fall below what is expected to be consumed during nursing by the calf, it can be considered an economic loss.

Understanding factors influencing lower-than-expected milk yields is crucial for improving the sustainability of cow-calf contact systems. Calf sex and lactation number were sources of variability in milk yield of cows with full-time cow-calf contact (Mutua and Haskell, 2021). There can also be issues with milk let-down in cows that nurse their calves, resulting in greater residual milk remaining in the udder after machine milking (de Passillé et al., 2008). Milk ejection requires the release of oxytocin locally in the mammary gland or peripherally, which may be impaired if the sympathetic nervous system is activated due to stress around the time of milking (Bruckmaier and Blum, 1998). Stress may arise from a poor human-animal relationship (Hemsworth et al., 2000), resulting in restlessness at milking (stepping and kicking by the cow) that may negatively affect milk yield (Sutherland and Dowling, 2014; Neave et al., 2022b). Milking procedures can also affect milk yield of cows, such as teat preparation to stimulate milk let-down (Sandrucci et al., 2007). However, these possible explanations for low machine milk yields have yet to be explored in cows in dam-rearing systems. Thus, the objective of this study was to investigate whether cow behavior (stepping and kicking in the parlor), milking procedures (a.m. or p.m. milking period, duration of teat and udder preparation, frequency of teat and udder stimulation), and animal characteristics (parity, calf sex) could explain low machine milk yields in cows with full-time or part-time cow-calf contact in dam-reared systems. Herein, the term milk yield will refer to machine milk yield.

Seventy-two Danish Holstein dairy cows (30 primiparous, 42 multiparous) were enrolled in 6 blocks (12 cows per block, with a new block enrolled every 5 wk) from September 2021 to July 2022 and managed in accordance with the Danish Ministry of Environment and Food Act No. 474 (May 15, 2014) and approved by the Danish Animal Experiments Inspectorate (permit no. 2021–15–0201–00989). At 48 h after calving, cows were pseudo-alternately assigned in pairs to 1 of 3 treatments (n = 24/treatment; 4 cows/treatment per block): (1) full-time cow-calf contact (23 h/d): dams and calves were separated only during milking times; (2) part-time cow-calf contact (10 h/d): dams and calves were housed together between a.m. milking and p.m. milking; (3) no-contact: cows were separated from their calves 48 h after birth. Within treatment and block, treatments were balanced for parity (10 primiparous and 14 multiparous cows per treatment), but not for calf sex (13, 14, and 11 female calves, and 11, 10, and 13 male calves for full-time, part-time, and no-contact treatments, respectively; each pen always had at least 1 of each calf sex, except on 2 occasions when the full-time treatment pen in block 5 had 4 bull calves, and in block 6 had 4 female calves). Cows and their calves had to be healthy at the time of treatment assignment, the calf needed to suckle without assistance, and no twins or complications during calving were permitted. Sample size was selected to ensure sufficient power for a concurrent study assessing behavioral responses to weaning depending on treatment (Neave et al., 2024).

Full- and part-time cows and their calves were housed in groups of 4 in straw-bedded pens (7.5 × 9 m), provided a TMR twice daily, as well as water, for ad libitum intake, and milked twice daily at 0500 and 1530 h in a double 12 parallel milking parlor (SAC, A/S S.A. Christensen & Co); milking vacuum was set to 39:39.5 pulsation ratio and 60 pulses/min, and clusters automatically detached when milk flow was below 0.40 kg/min. After the p.m. milking, part-time cows were housed in another barn where they had no contact with their calves. Part-time cows were reunited with their calves after the a.m. milking. Full-time cows were reunited with their calves directly after each milking.

No-contact cows were housed in a separate barn with no visual or auditory contact with their calves. They were housed in a pen of 8 other cows (4 of which were nonexperimental) with 12 lying stalls equipped with mattresses, fed the same TMR as full- and part-time cows twice daily in computerized feed bins, and milked twice daily at 0530 and 1600 h in the same parlor as full-time and part-time cows.

Full- and part-time cows were milked first on the right and left sides of the parlor, respectively; no-contact cows were milked second on the right side of the parlor. The remaining 8 milking stalls on each side of the parlor were filled with cows from other pens. Treatments experienced the same milkers within a given milking, but milkers differed between a.m. and p.m. shifts. Milkers were instructed never to administer oxytocin. Standard milking procedure guidelines were to prepare and begin milking cows in sets of 4, where cows first had their teats disinfected with a pre-milking iodine solution, then had their teats wiped, and finally had the milking cluster attached; however, adherence to these procedures was not mandated and varied as observed from recorded video (resulting in some very short and very long preparation times). Manual teat and udder stimulation was at the discretion of the milker, and milkers could make their own individual decisions regarding teat cup application and removal; in most cases, all teat cups were applied and were left to automatically detach. Cows and milkers were observed using 2 cameras (GoPro Hero7, GoPro Inc.) on 2 d (a.m. and p.m. milking each day) during wk 4 and 6 of the treatment period, for a total of 8 milkings per cow (mean ± SD DIM; wk 4: 33.9 ± 7.3 and 36.1 ± 6.9; wk 6: 45.8 ± 5.7 and 48.4 ± 5.6, for each day within week, respectively).

Behavior of cows and milkers (milking procedures) were measured from video recordings over the entire milking process, divided into 5 segments: preparation, attachment, milking, cluster detaching, and reattachment (if performed). For each segment, frequency of cow behaviors (steps and kicks, including kicks resulting in the cluster detaching), frequency of teat or udder stimulation, and duration of the segment were recorded (described in Table 1) using BORIS software (Friard and Gamba, 2016). Two observers achieved interobserver reliability (8 cows for full milking process; Cohen's kappa >0.9, calculated in BORIS with 1 s threshold). Milk yields at each milking were recorded from the in-parlor monitor (SAC, A/S S.A. Christensen & Co.); any missed or zero yields were verified in the online database. Blinding was not possible because cows from a particular treatment were always in the same milking stalls.

**Table 1:** Ethogram of cow behavior and milking procedures scored during each milking (n = 70 cows)

Variable	Description
Milking segment	
Preparation (min)	From moment milker initially touches any part of the cow until milker touches the cluster to begin attachment. Includes waiting time in between teat or udder stimulation, teat disinfecting, and teat wiping.
Attachment (min)	From moment milker touches the cluster until the cluster is attached to all 4 teats and the milker's hand is no longer touching the cluster, cow, udder, or hose.
Milking (min)	From end of attachment until the cluster vacuum stops (cluster no longer hangs vertically from the teats, but may remain on the teats).
Cluster detaching (min)	From moment milking ends until the cluster completely detaches from all 4 teats (may be removed by milker).
Reattachment (min)	Only if milking has stopped. From moment milker touches the cluster and re-attaches (or repositions cluster under the udder) resulting in the cluster hanging vertically from the teats and milking resumes. Reattachment was performed at discretion of milker after assessing udder fill.
Cow behavior	
Step (no.)	Any movement of either hind hoof, of any height, excluding a kick.
Kick (no.)	Forceful and rapid movement of either hind leg backward or forward, or toward the milking cluster.
Kick off cluster (no.)	Kick resulting in the milking cluster detaching from all 4 teats.
Milking procedure	
Teat or udder stimulation (no.)	Milker places a hand on any of the teats or udder. Milker may rub the udder or strip the teat. Includes both touching and manual stimulation of teat or udder.
Cluster re-attached (no.)	Only if milking has stopped. Milker touches the cluster and re-attaches (or repositions cluster under the udder) resulting in the cluster hanging vertically from the teats and milking resumes.
Cluster removed (no.)	Milker removes the cluster from all 4 teats.

Data were summarized as the sum number of steps and kicks during the milking process (preparation + milking segments, excluding during cluster detachment because this was not contributing to milk yield), as well as the duration of each milking segment, frequency of stimulation by the milker, and frequency of cluster detaching and re-attachments. The number of steps and kicks within each milking segment was summarized separately. Because the duration of each milking segment differs for each cow, the number of steps and kicks for each cow was calculated as total movements per minute over the entire milking process (excluding steps and kicks during cluster detachment, and excluding duration of cluster detachment). One no-contact and one part-time cow were removed from the experiment for health issues. Some observations were missing due to lost video files (19 observations), cow not being visible in the video (6 observations), or camera failure part-way through milking (20 observations). One extreme outlier for preparation duration (9.3 min) was removed. The final sample size was 70 cows (n = 24 full-time, 23 part-time, 23 no-contact), with at least 3 of 4 complete a.m. and p.m. milking observations for each cow.

All statistical analyses were performed in SAS Studio (OnDemand for Academics, SAS Institute Inc.), with cow (n = 70) as the experimental unit. Significance level was declared at *P* ≤ 0.05, and tendencies at *P* ≤ 0.10. The response variables of milk yield and total movements per min over the entire milking process approximated a normal distribution (Shapiro-Wilk test, W ≥0.85), verified with visual examination of histograms and model residuals. Model fit was assessed using Akaike information criterion and compared with null model with likelihood ratio test. Pair-wise comparisons among treatments were assessed and adjusted using Tukey-Kramer tests. To examine our primary study hypothesis, a mixed linear regression model (PROC MIXED) tested whether milk yield was affected by treatment (full-time, part-time, or no-contact), milking period (a.m. or p.m.), parity (primiparous or multiparous), total movements per minute over the entire milking, preparation duration, stimulation frequency, and their 2-way interactions with treatment. Additional fixed effects tested were DIM and calf sex (male or female). After backward elimination of nonsignificant factors, the final model included treatment, milking period, parity, total movements per minute over the entire milking, stimulation frequency, treatment × milking period, and treatment × parity. Cow within pen within block were random effects, accounting for repeated milking observations with compound symmetry covariance structure.

Next, we performed a further exploratory analysis of factors that might affect restlessness of cows at milking, building an identical model to that described above except that total movements per minute over the entire milking process was the response variable, and milk yield was included as an additional fixed effect. After backward elimination of nonsignificant factors, the final model included treatment, milking period, stimulation frequency, and treatment × stimulation frequency.

Similar to previous studies (Barth, 2020; Mutua and Haskell, 2021), there was large variability in daily milk yields in cows with full-time (mean ± SD: 11.6 ± 5.5 L/d, range: 3.0–23.4 L/d), part-time (26.1 ± 8.2 L/d, range: 5.5–41.0 L/d), and no calf contact (37.3 ± 9.7 L/d, range: 19.5–57.0 L/d). Cow-calf contact treatment affected machine milk yield, depending on milking period (*F*_2,67_ = 156.7, *P* < 0.001; Figure 1a). No-contact cows had the highest milk yields, delivering less milk during p.m. than a.m. milking (17.3 and 19.9 ± 0.7 L, respectively; *t*_1,67_ = 4.7; *P* < 0.001). Full-time cows had the lowest milk yields, which were similar at a.m. and p.m. milking (6.1 and 6.0 ± 0.7 L, respectively; *t*_1,67_ = 0.1; *P* = 1.0). Part-time cows had lower milk yield at p.m. than a.m. milking (*t*_1,67_ = 23.4; *P* < 0.001); p.m. milk yield was comparable to full-time cows (6.4 ± 0.7 L), and a.m. milk yield was comparable to no-contact cows (19.6 ± 0.7 L). This result is likely due to a lack of milk removal by the calves overnight (in the no-suckling period), a longer time period between p.m. and a.m. milking, or cows may have been anticipating reunion with their calves after a.m. milking, leading to increased oxytocin release. Similarly, Barth (2020) reported about 13 L/d less milk yield from part-time cows permitted to suckle only during the nighttime, compared with cows with no calf contact.

**Figure 1:**
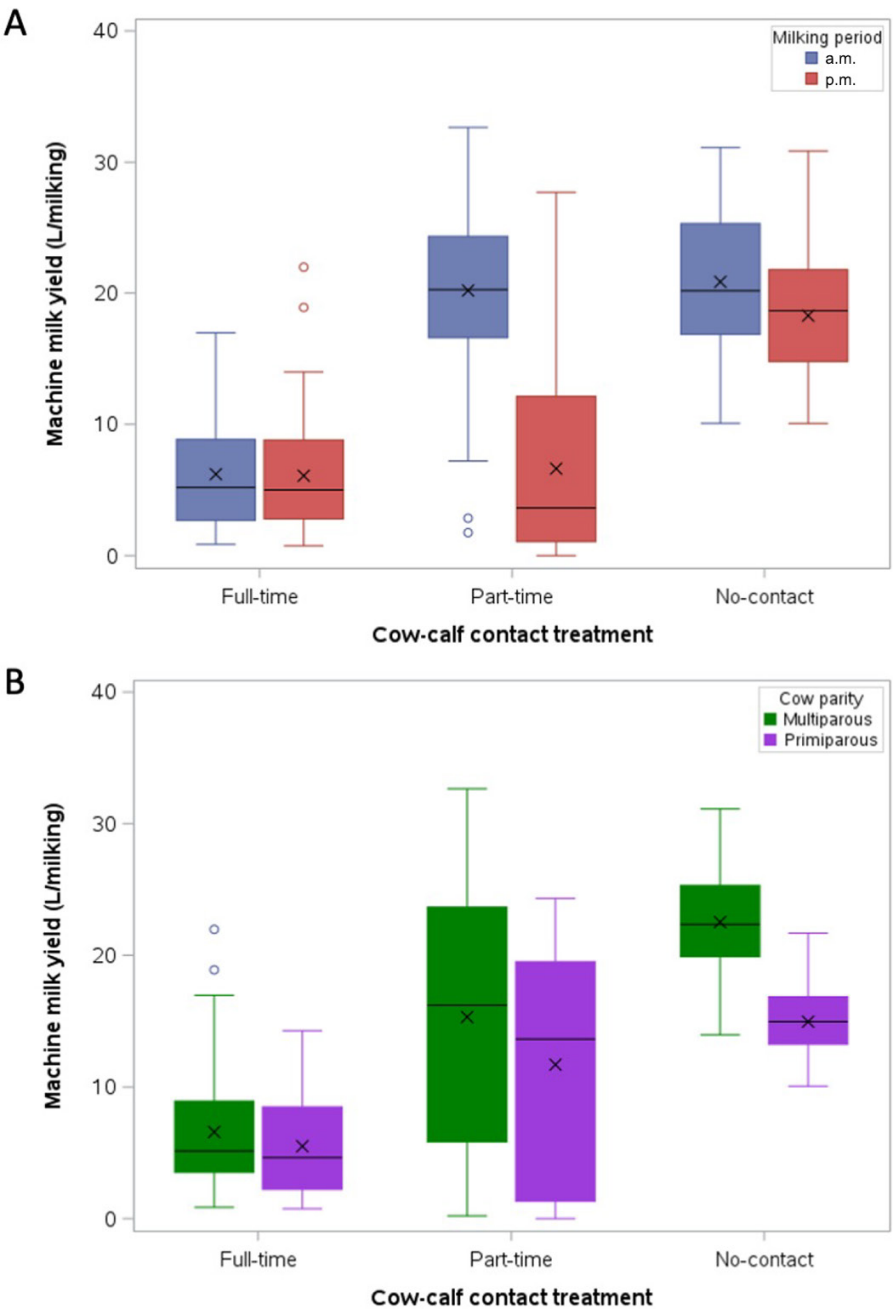
Boxplots of the machine milk yield (L per milking) for each cow-calf contact treatment (full-time, 23 h/d; part-time, 10 h/d in daytime only; or no cow-calf contact) depending on (A) milking period (a.m. or p.m.), and (B) parity (primiparous or multiparous). Cows (n = 24, 23, and 23 for full-time, part-time, and no-contact, respectively) were observed at 8 milkings over wk 4 and 6 of the treatment period. Each boxplot contains the following elements: first and third quartiles (box), median (horizontal black line inside box), mean (black × inside box), minimum and maximum (whiskers, vertical lines), and outliers (open circles, if applicable).

Full-time cows had lower daily milk yield than expected (about 12 L/d). Other studies providing 24 h/d cow-calf contact reported 13 L/d (Barth, 2020) and 17 L/d (Wenker et al., 2022), but their reference group of no-contact cows produced less (29 L/d) than in our study (about 37 L/d). The difference in daily milk yield between our full-time and no-contact cows (25 L/d) is likely not fully explained by milk consumption by the calf. It is difficult to determine how much milk suckling calves consume in a 24-h period, but estimates of milk intake by calves in restricted suckling systems indicate 9 to 12 L/d (de Passillé et al., 2008; Fröberg et al., 2008), and between 10 and 14 L/d by calves fed ad libitum from an artificial teat (Jasper and Weary, 2002; Frieten et al., 2017). However, not all studies report lower milk yields in cows that nurse their calves (Meagher et al., 2019). Other factors (some of which we explored herein) are likely leading to these lower-than-expected milk yields in cows after a period of calf contact.

Cow parity is known to affect machine milk yield early in lactation (Masía et al., 2020); this was also the case in our cow-calf contact system, but depended on treatment (*F*_2,64_ = 6.5; *P* < 0.01; Figure 1b). Multiparous cows yielded (or tended to yield) more milk than primiparous cows in both no-contact (22.5 ± 0.9 and 14.7 ± 1.1 L/milking, respectively; *t*_1,64_ = 5.7; *P* < 0.001) and part-time treatments (15.0 ± 0.9 and 11.1 ± 1.0 L/milking, respectively; *t*_1,64_ = 2.8; *P* = 0.06). However, no difference in machine milk yield was detected between full-time primiparous and multiparous cows (5.6 ± 1.0 and 6.5 ± 0.9 L/milking, respectively; *t*_1,64_ = 0.7; *P* = 0.98); this contrasts 2 previous studies (Barth, 2020; Mutua and Haskell, 2021) that reported greater daily milk yield in multiparous versus primiparous cows with full-time calf contact. A possible explanation may be that cows in our study experienced cow-calf contact (and temporary separations for milking) for the first time, which may have affected milk yield, while the multiparous cows in previous studies had prior experience. Alternatively, calves (including alien calves) in the full-time treatment may have consumed more milk from multiparous versus primiparous cows. These hypotheses remain to be tested to understand parity differences (or lack thereof) in machine milk yield of cows with calf contact.

Calf sex did not affect machine milk yield (12.6 and 12.5 ± 0.6 L/milking, for cows rearing male and female calves, respectively; *F*_1,63_ = 0.04; *P* = 0.8). A previous study reported that cows with full-time access to their male calves had lower milk yield than cows with female calves (Mutua and Haskell, 2021). Higher birthweight and growth demands by male offspring are reported in other species (sheep: Festa-Bianchet et al., 1996; deer: Landete-Castillejos et al., 2005); indeed, ADG of our male calves from full-time cows was numerically greater than female calves at wk 7 of the treatment period (1.2 vs. 0.9 ± 0.4 kg/d, respectively). Presumably greater milk consumption by male calves supported this higher growth rate, but similar milk yields between cows with male versus female calves suggests that calf sex was not a main driver of variability in milk yield in this study.

Machine milk yield was negatively influenced by the frequency of teat or udder stimulation (estimate: −0.72 ± 0.3 L/milking; *F*_1,419_ = 4.9; *P* = 0.03) but did not depend on treatment (*F*_2,417_ = 1.4, *P* = 0.3), likely because stimulation frequency was very similar across treatments (mean ± SD; 2.2 ± 0.6, range 0 to 5 stimulations/milking). We did not distinguish the type of stimulation (touching versus manual stimulation of teats), which may have different effects on the animal and milk yield. Neither preparation duration (*F*_1,416_ = 0.1, *P* = 0.85), nor its interaction with treatment (*F*_2,416_ = 0.10, *P* = 0.90), affected milk yield, likely because mean preparation durations were similar across treatments (mean ± SD; 1.3 ± 1.0, range 0.3 to 4.3 min/milking). Similarly, preparation time did not affect milk yield of Danish Holstein cows (Rasmussen et al., 1992), or other breeds (Weiss and Bruckmaier, 2005), although the cows in these studies had no calf contact. A longer preparation time of 1.5 min was recommended for cows with low udder fill (Weiss and Bruckmaier, 2005), which could be beneficial for cow-calf contact systems.

Total movements over the entire milking process (i.e., stepping and kicking behavior) negatively influenced milk yield, but this effect was minor (estimate: −0.16 ± 0.07 L/milking; *F*_1,419_ = 4.7; *P* = 0.03), and did not depend on treatment (*F*_2,417_ = 0.9, *P* = 0.42). This suggests that restlessness behavior in the milking parlor may contribute to lower milk yield, but could not explain the lower milk yields of cows with calf contact. Previous studies have also found that a greater frequency of stepping and kicking in the milking parlor was associated with lower milk yields (Hedlund and Løvlie, 2015; Marçal-Pedroza et al., 2020; Neave et al., 2022b).

Restlessness behavior in the parlor is undesirable, so we also explored factors that may influence this behavior. Total number of steps and kicks over the entire milking process were highly variable among individual cows (range; full-time: 6 to 251; part-time: 6 to 136; no-contact: 6 to 145 steps and kicks). Total movements per minute over the entire milking process were more frequent at p.m. than a.m. milking regardless of treatment (5.3 and 6.1 ± 0.3 movements/milking; *F*_1,69_ = 19.8, *P* < 0.001). Total movements per minute were also affected by treatment, depending on stimulation frequency (*F*_2,420_ = 4.8, *P* < 0.01). No-contact cows moved more frequently with greater stimulation frequency compared with full- and part-time cows (slope = 1.4 ± 0.5 movements/stimulation; *t*_1,420_ = 2.8, *P* < 0.01), while full- and part-time cows showed no relationship between movements and stimulation frequency (slope = −0.04 ± 0.5 movements/stimulation; *t*_1,420_ = 0.08, *P* = 0.94). Stepping and kicking in the parlor can be a sign of a negative emotional response to the milking process, discomfort, or other stressors in the environment (Rushen et al., 2001). The no-contact cows may have experienced discomfort due to fuller udders, leading to stepping and kicking in response to more frequent stimulation of the udder or teats. Full- and part-time cows stepped and kicked as frequently as no-contact cows (as noted above), but the lack of relationship with stimulation frequency suggests they showed restless behaviors for different reasons. For example, Roadknight et al. (2022) reported more stepping and kicking at p.m. milking by pastured cows with access to their calves only during the nighttime. These authors speculated that restlessness was a negative response to prolonged separation from their calves, or in the case of our study, may be due to frustration or anticipation related to the temporary separation from their calves during milking (both full- and part-time cows) or upcoming overnight separation from their calves (part-time cows only). Alternatively, both full- and part-time cows could be experiencing udder or teat discomfort due to overmilking of one or several udder quarters, leading to increased stepping and kicking at p.m. milking. Moreover, different milkers at a.m. and p.m. milkings could elicit more or less restless behaviors in cows (through stimulation or preparation techniques) depending on their attitudes and actions toward cows (Waiblinger et al., 2002).

In conclusion, cows with prolonged calf contact, especially full-time, showed lower machine milk yields than can be expected due to milk consumption by the calf. Cows with part-time calf contact (separated overnight) had improved milk yield at a.m. milking, likely owing to the no-suckling period. Lower machine milk yields in cows with calf contact could not be explained by differences in cow parity, calf sex, milking procedures (stimulation frequency; preparation duration), or stepping and kicking (restlessness) behavior at milking. All cows showed more restless behavior at p.m. milking, but this was unrelated to stimulation frequency in full- and part-time cows. Future research should investigate possible reasons and solutions for low yield or milk ejection issues in cows with calf contact, such as longer preparation times, or presence of the calf at milking as in automated milking systems.
